# Epidemiology of hospital-acquired bloodstream infections in haemato-oncology patients in Geneva, Switzerland

**DOI:** 10.1007/s15010-025-02524-w

**Published:** 2025-04-09

**Authors:** Aleece MacPhail, Marie-Noëlle Chraïti, Aude Nguyen, Gaud Catho, Loic Fortchantre, Marie-Céline Zanella, Véronique Camus, Stavroula Masouridi-Levrat, Dionysios Neofytos, Zoe McQuilten, Stephan Harbarth, Niccolò Buetti

**Affiliations:** 1 Infection Control Program and WHO Collaborating Centre, Geneva University Hospitals and Faculty of Medicine, Geneva, Switzerland; 2 Department of Infectious Diseases, Monash Health, Melbourne, Australia; 3 School of Public Health and Preventive Medicine, Monash University, Melbourne, Australia; 4 Division of Infectious Diseases, Faculty of Medicine, Geneva University Hospitals, Geneva, Switzerland; 5 Infectious Diseases Division, Central Institute, Valais Hospital, Sion, Switzerland; 6 Division of Haematology, Faculty of Medicine, Geneva University Hospitals, Geneva, Switzerland; 7 Infection Antimicrobials Modeling Evolution (IAME), INSERM, Université Paris-Cité, Paris, U 1137 France

**Keywords:** Bloodstream infection, Epidemiology, Hospital-acquired infection, Haemato-oncology, Catheter associated bloodstream infection, Antimicrobial resistance

## Abstract

**Background:**

Hospital-acquired bloodstream infections (HA-BSI), including catheter-associated bloodstream infections (CABSI), cause preventable harm in haemato-oncology patients but surveillance data are limited.

**Methods:**

We performed a retrospective cohort study using prospectively collected data in a large hospital network in Switzerland from 2017–2022. Incidence, source, and microbiology of HA-BSI were compared between (1) haematology patients with acute leukaemia or allogeneic stem cell transplantation (2) oncology patients with solid tumour or lymphoma, and (3) general medical patients. No routine quinolone prophylaxis was prescribed.

**Results:**

We included 320,058 patient-days and 201,081 catheter-days across two haematology, two oncology and nine non-COVID-19 general medical wards. 669 HA-BSI occurred in 547 individual patients. In haematology patients, HA-BSI incidence was 9.1/1000 patient-days (95% CI 8.2–10.3). 224/299 (75%) of episodes were “unknown/other” source. Low virulence Gram-positive organisms (coagulase-negative staphylococci, *viridans Streptococci*, enterococci) accounted for 232/378 (61%) HA-BSI organisms and 46/52 (88%) CABSI organisms. Compared to oncology and general medical patients, haematology patients had higher HA-BSI incidence, but a smaller proportion of infections caused by virulent organisms (Gram-negative bacteria, *Staphylococcus aureus*, *p* < 0.01).

**Conclusions:**

In haematology patients, HA-BSI are less commonly caused by virulent Gram-negative organisms or *Staphylococcus aureus* compared to solid tumour and general medical patients, in the absence of quinolone prophylaxis.

**Supplementary Information:**

The online version contains supplementary material available at 10.1007/s15010-025-02524-w.

## Background

Hospital-acquired bloodstream infections (HA-BSI) are an important cause of morbidity and mortalityamong hospitalised patients with solid tumour and haematological malignancies [1, 2]. HA-BSI incidence and HA-BSI associated mortality are both higher in haemato-oncology units than in the general hospital population [1, 2]. An understanding of the epidemiology, sources of infection, and microbiology of HA-BSI is important to guide prevention efforts, inform empirical treatment, and benchmark hospital performance.

HA-BSI include catheter associated bloodstream infections (CABSI), secondary bloodstream infections (BSI) related to infections at another site, and HA-BSI of unknown source. In haemato-oncology patients experiencing profound neutropenia, BSI of “unknown/other source” may represent translocation of endogenous flora across an inflamed oral or gastrointestinal mucosa, also known as mucosal barrier injury bloodstream infections (MBI-BSI).

Much of the existing data describing HA-BSI are drawn from hospital surveillance. However, surveillance systems generally have a strong focus on CABSI at the expense of other HA-BSI types [3–5]. In haemato-oncology patients, accurate identification of CABSI can be difficult, and case definitions are associated with high rates of misclassification [4–7]. Some researchers have proposed surveillance of all HA-BSI, but this is not yet widespread practice [4, 5]. Other epidemiological data are sourced from observational cohort studies. However, existing reports typically include only neutropenic patients, or cohorts receiving quinolone prophylaxis [8, 9], a practice that is gradually changing in many parts of the world [8, 10, 11].

In our institution, no routine quinolone prophylaxis is used for prevention of bacterial infection during chemotherapy-induced neutropenia and pre-engraftment of allogeneic stem-cell transplantation. All hospital-onset BSI are prospectively investigated by a dedicated team of infection prevention and control (IPC) specialists [12]. We used these data to conduct a retrospective cohort study of HA-BSI in cancer patients from 2017 to 2022. We aimed to describe the incidence, source, and microbiology of all HA-BSI in haematology and oncology patients, using a cohort of general medical patients as a comparator group.

## Methods

### Study design

We performed a retrospective cohort study of patients with HA-BSI admitted to haematology, oncology and general medical wards at Geneva University Hospitals (HUG) from January 1, 2017 to December 31, 2022.

### Setting

HUG is a 2,100-bed tertiary hospital network in Geneva, Switzerland that receives approximately 60,000 hospital admissions per year. Dedicated haematology and oncology services admit patients with cancer diagnoses. Patients with acute leukaemia and recipients of allogeneic stem cell transplant (alloSCT) are cared for under the haematology service in two dedicated wards. Patients with solid organ cancers and lymphoma share two dedicated wards. No autologous stem cell transplants are performed at HUG.

### Inclusion criteria

We included all hospitalized adult patients (≥ 18 years old), admitted to a haematology, oncology, or general medical ward, with at least one HA-BSI between January 1, 2017 and December 31, 2022. Dedicated COVID-19 medical wards were excluded from the general medical group.

In the case of repeat positive blood cultures with the same causative organism, occurring within 14 days, only the first episode was included unless investigation revealed distinct sources of infection.

### Patient groups

Patients were classified into three groups according to their ward of admission: haematology group (admitted to one of two acute leukaemia and allogeneic stem cell transplant wards), oncology group (admitted to one of two solid cancer and lymphoma wards), and general medicine group (admitted to one of nine non-COVID-19 general medical wards).

The nine medical units consisted of “general medicine” or “internal medicine” patients and did not include patients admitted under surgical, intensive care (ICU), emergency, paediatric, psychiatric, or rehabilitation units.

### Outcomes and definitions

The primary outcomes were HA-BSI defined as culture of a pathogen from blood culture 48 h after the beginning of hospital admission or meeting one of the following criteria: readmission < 10 days after discharge from a previous admission; care in an ambulatory dialysis or haemato-oncology unit < 30 days prior; infection related to a surgery < 30 days prior; or infection related to surgery with implantation of a prosthesis < 365 days prior. Common skin contaminants as defined by the ECDC and NHSN organisms lists [13, 14] were included only if the patient had at least one sign or symptom infection (e.g. fever > 38^o^C, chills, hypotension) and two positive blood culture results from two separate blood samples (i.e. two blood draws from separate sites and/or separate times) within 48 h. Institutional guidelines for blood culture collection require two sets of blood cultures be taken prior to antibiotics.

HA-BSI were classified as either CABSI, secondary BSI relating to infection at another site, or BSI of unknown/other source. For CABSI, we included cases meeting ECDC criteria for Catheter Related Infection (CRI3), or ECDC Hospital-acquired Bloodstream Infection of Central/Peripheral Catheter Origin (ECDC-C-CVC/P). CRI3 is a more stringent definition requiring microbiological confirmation of catheter source (supplementary methods). In cancer patients at HUG, microbiological confirmation of CABSI is established through routine catheter tip culture of centrally dwelling vascular catheters, permitting application of the CRI3 criteria. Further details regarding case definition are presented in the supplementary methods. Infections with onset outside of our institution were not captured.

### Microbiological testing

Blood samples were processed using BACTEC™ (Becton, Dickinson Microbiology Systems). Isolates were identified by standard microbiological techniques including culture and Matrix-assisted laser desorption/ionisation time-of-flight mass spectrometry (MALDI-TOF). Susceptibility profiles were defined according to European Committee on Antimicrobial Susceptibility Testing (EUCAST) breakpoints.

### Data sources

All hospital-onset BSIs are investigated by the HUG IPC team as previously described [12], and those meeting ECDC criteria are recorded into a hospital database. Data collected include ward of acquisition, date of onset, relevant surveillance criteria met, catheter type for CABSI, micro-organism identified, and resistance profile for resistant organisms including: methicillin-resistant *Staphylococcus aureus* (MRSA), carbapenemase-producing Enterobacteriaceae (CPE), vancomycin-resistant enterococci (VRE), multidrug-resistant *Pseudomonas aeruginosa* (MDR-PsA, defined as resistant to ≥ 1 antibiotic in ≥ 3 antimicrobial categories) and carbapenem resistant *P. aeruginosa* (CR-PsA)).

Additional data were extracted from the hospital’s electronic health information system. Extracted data were total patient-days of admission per ward and bed occupation during the study period, and catheter characteristics (type, date of insertion and date of removal).

### Vascular catheters

Vascular catheter types were classified into four groups: 1) short-term non-tunnelled central venous catheters (hereafter referred to as “CVC”); 2) totally implantable venous access devices (TIVAD), e.g. Port-a-cath™; 3) peripheral venous catheter (PVC); 4) other catheter types including arterial catheters, dialysis catheters, and peripherally inserted central catheters (PICC-lines). In our institution, CVC are preferentially used on the haematology ward, and TIVAD are preferentially used on the oncology ward. Tunnelled CVCs such as Hickman catheters are not routinely used at the HUG, and no midline catheters are used. Institutional catheter care procedures are described in the supplementary methods.

### Statistical analysis

Descriptive analysis was performed. HA-BSI incidence, source and microbiological aetiology were compared between each group (haematology, oncology, and general medicine). Results were reported as n (%), mean (standard deviation [SD]) and median (interquartile range [IQR]) as appropriate. Incidence density per patient-days (HA-BSI) and catheter-days (CABSI) were calculated with 95% confidence interval (95% CI) assuming Poisson distribution using Newton’s method. Group comparisons between admitting units were made using Chi-square tests for equal proportion, students t-test or ANOVA for normally distributed outcomes, and Wilcoxon-Mann-Whitney or Kruskal-Wallis tests otherwise.

Catheter use (CVC, PVC and TIVAD) was reported as catheter-days, and device utilisation ratio (DUR) was calculated as [patient-days with catheter in situ]/[total patient-days]. For TIVADs, only catheter-days during which a device was accessed were reported.

### Ethics

Prospective surveillance of HA-BSI was conducted as part of the routine quality improvement activities of our infection control program. Analysis was performed on anonymized non-genetic surveillance data, and ethical consent was not required according to the Swiss law for research on humans (Article 33, Paragraph 2, Human Research Act).

## Results

There were 320,058 patient-days, 201,081 catheter-days, and 50,228 hospital admissions over the study period, across two haematology, two oncology and nine non-COVID-19 general wards providing acute medical care (Table 1). The most commonly used catheter type in haematology patients was short-term non-tunnelled CVC (DUR 0.71); in oncology patients the most commonly used catheter types were TIVAD (DUR 0.33) and PVC (DUR 0.29); and in general medical patients the most commonly used catheter type was PVC (DUR 0.75, Table 1).

**Table 1:** Patient and line characteristics of included patients, 2017–2022

	Haematology	Oncology	General medicine
Total admissions	1,300	4,513	44,415
Patient-days	32,591	34,950	252,517
Catheter-days			
CVC	23,334	851	6,185
PVC	2,150	9,936	190,018
TIVAD*	2,006	11,513	7,877
Device utilisation ratio			
CVC	0.71	0.02	0.02
PVC	0.07	0.29	0.75
TIVAD*	0.06	0.33	0.03
% of all admissions ≥ 1 catheters inserted (CVC/PVC) or accessed (TIVAD)			
CVC	52.7	1.6	2.0
PVC	45.1	51.0	71.0
TIVAD*	16.9	37.2	2.3

CVC short-term non-tunnelled central venous catheter PVC peripheral venous catheter TIVAD totally implantable vascular access device. Device utilisation ratio = total catheter-days/total patient-days

*Only days in which a TIVAD was accessed are included

669 HA-BSI among 547 individual patients were reported, including 112 polymicrobial infections and a total of 822 individual organisms isolated (supplementary Fig. 1). Of all HA-BSI, 430/669 (64%) occurred in male patients. Patients developing HA-BSI were slightly younger in the haematology unit (median 59 years, IQR 46–66 years), and oncology unit (median 60 years, IQR 52–73 years), compared to the general medical unit (median 66 years, IQR 55–77 years, *p* < 0.01).

### HA-BSI and CABSI incidence

Incidence density of HA-BSI (per 1,000/patient-days, 95% CI) was as follows: haematology 9.1 (8.2–10.3), oncology 2.6 (2.1–3.2), general medicine 1.1 (1.0-1.3). CABSI incidence/1,000 catheter-days across different catheter types is reported in Table 2 and was highest for haematology patients with CVC (16.7, 11.9–22.8).

**Table 2:** Incidence of HA-BSI and CABSI

	Haematology HA-BSI *n* = 299 CABSI *n* = 45		Oncology HA-BSI *n* = 91 CABSI *n* = 20		General medicine HA-BSI *n* = 227 CABSI *n* = 52	
		(95% CI)		(95% CI)		(95% CI)
HA-BSI /1000 patient days	9.1	8.2–10.3	2.6	(2.1–3.2)	1.1	(1.0–1.3)
CABSI per catheter days						
CLABSI /1000 CVC-days	1.7	(1.2–2.3)	2.4	(0.3–8.5)	1.3	(0.6–2.6)
PVC-BSI/1000 PVC-days	0.5	(0.0–2.6)	0.1	(0.0–0.6)	0.1	(0.1–0.2)
TIVAD- BSI/1000-TIVAD access days*	1.0	(0.1–3.6)	1.3	(0.7–2.2)	1.3	(0.6–2.3)
Patient demographics of patients who developed HA-BSI						
Male sex (%)	197	(65.9%)	53	(58.2%)	180	(64.5%)
Age, median years (IQR)	59	(46–66)	60	(52–73)	66	(55–77)

HA-BSI Hospital-acquired bloodstream infection CABSI Catheter-associated bloodstream infection

CLABSI Central line-associated bloodstream infections PVC-BSI Peripheral venous catheter-associated bloodstream infection TIVAD-BSI totally implantable vascular access device associated bloodstream infection

CVC short-term non-tunnelled central venous catheter PVC peripheral venous catheter TIVAD totally implantable vascular access device IQR interquartile range

*Only days in which a tunnelled catheter was accessed are included

### HA-BSI source

**Fig. 1:**
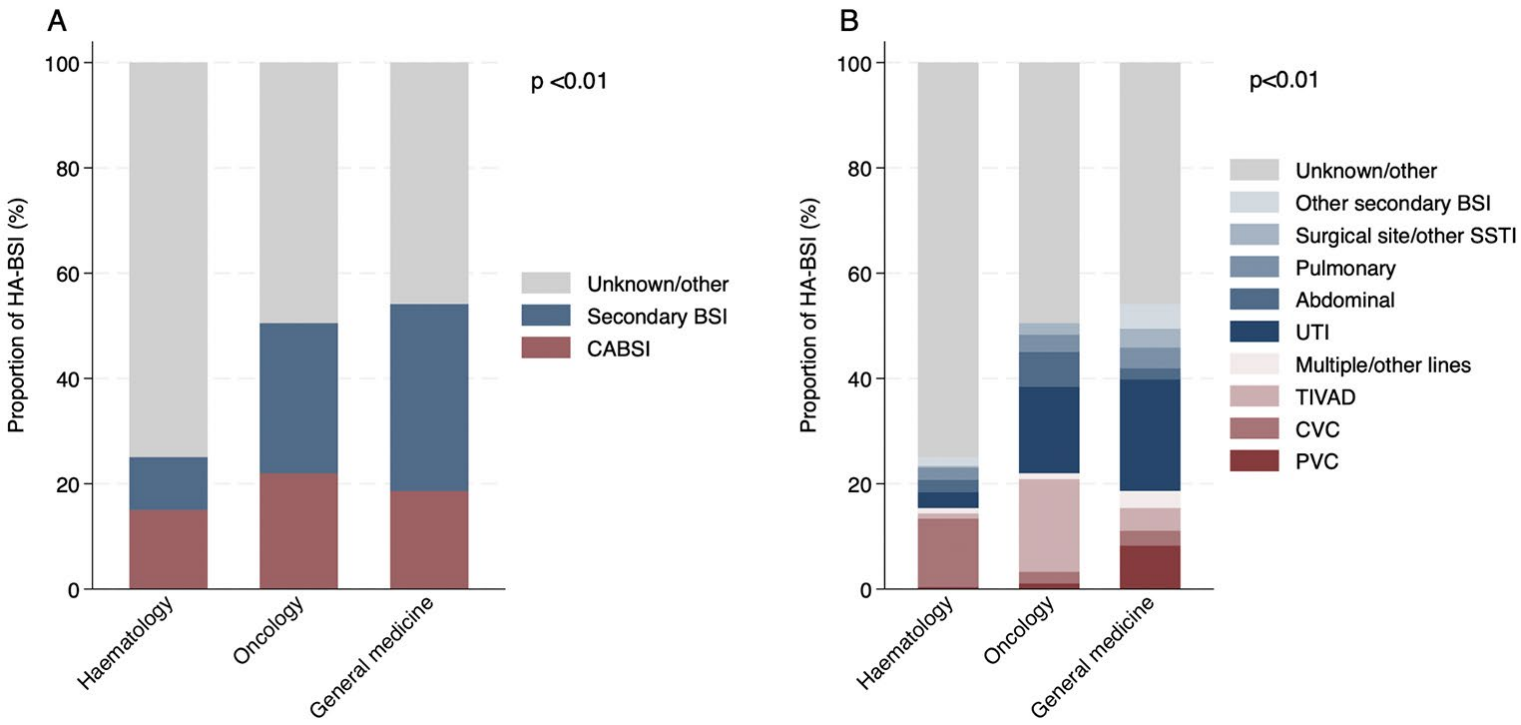
Comparison of HA-BSI sources across patient groups **A**: HA-BSI classification. **B**: Source subtype. HA-BSI hospital-acquired bloodstream infection BSI bloodstream infection CABSI catheter associated bloodstream infection SSTI Skin and soft tissue infection UTI Urinary tract infection TIVAD Totally implantable vascular access device CVC central venous catheter PVC peripheral venous catheter

Distribution of HA-BSI source differed between haematology, oncology, and general medicine patients (Fig. 1).

A total of 117 CABSI were identified, of which 58/117 (50%) were microbiologically confirmed by CRI3 criteria. CABSI accounted for a similar proportion of all HA-BSI across the three groups: haematology 45/299 (15%); oncology 20/91 (22%); general medicine 52/279 (19%, *p* = 0.25).

Secondary BSI were less common in haematology patients compared to other groups: haematology 30/200 (10%); oncology 26/91 (29%); general medicine 99/279 (35%, *p* < 0.01). This difference was most marked for BSI secondary to hospital-acquired urinary tract infection: haematology 9/299 (3%); oncology 15/91 (16%); general medicine 59/279 (21%, *p* < 0.01). HA-BSI secondary to skin and soft tissue infections (including surgical site) were also less common in haematology patients: 1/299 (0.3%); oncology 2/91 (2%); general medicine 10/279 (4%, *p* = 0.02). Secondary HA-BSI of confirmed abdominal source were more common in oncology patients than haematology or general medicine, but this did not reach significance: haematology 7/299 (2%); oncology 6/91 (7%); general medicine 6/279 (3%, *p* = 0.07). There was no difference in the proportion of HA-BSI of pulmonary source.

In contrast, haematology patients had the highest proportion of BSI of unknown/other source: haematology 224/299 (75%); oncology 46/91 (49%); general medicine 128/279 (46%, *p* < 0.01).

### Microbiology of HA-BSI

**Fig. 2:**
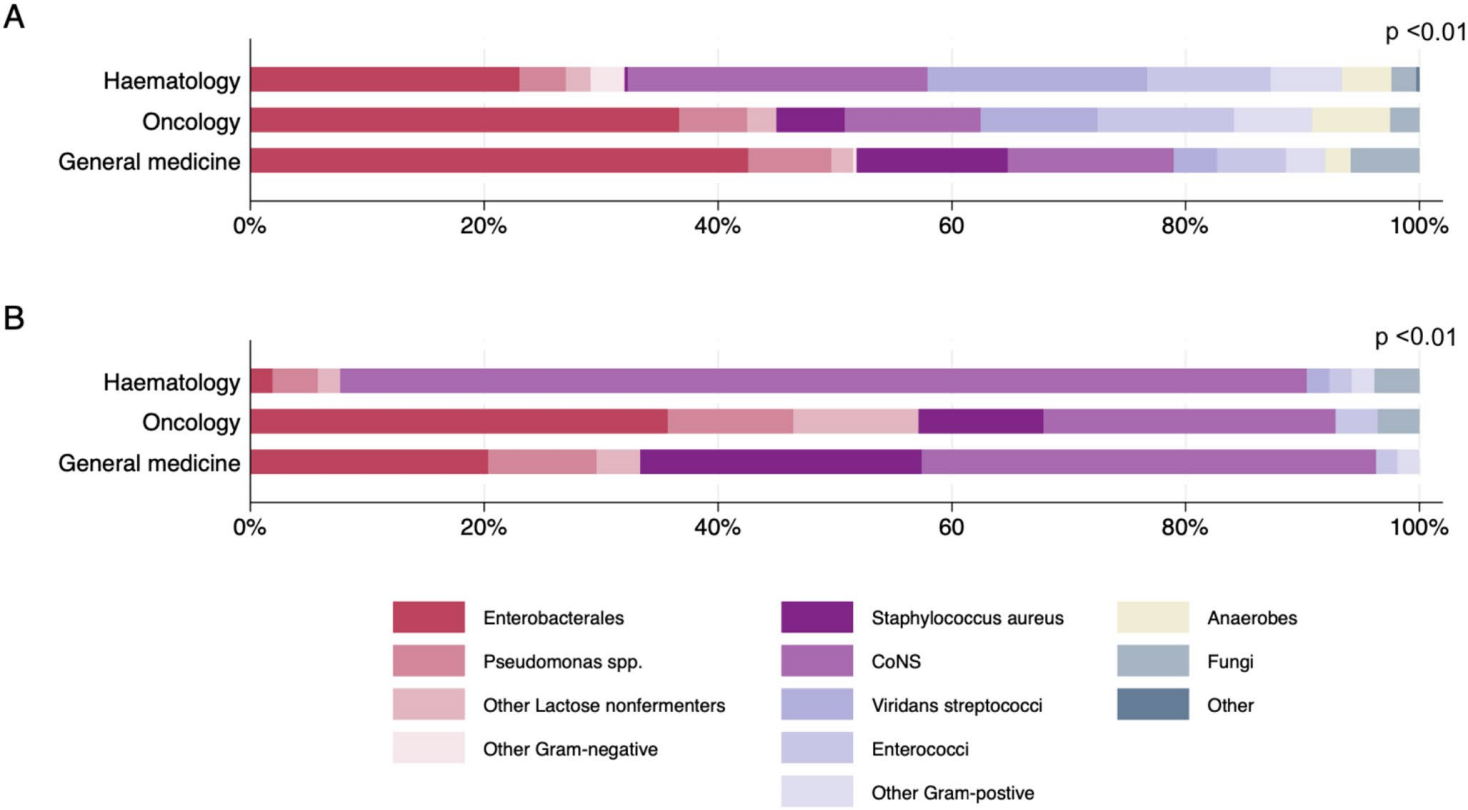
Microbiology of hospital-acquired bloodstream infections. **A**: Microbiology of all HA-BSI *n* = 669 episodes, 822 unique organisms isolated. **B**: Microbiology of CABSI *n* = 117 episodes, 134 unique organisms isolated. HA-BSI: Hospital-acquired bloodstream infection; CABSI: Catheter associated bloodstream infections; CoNS: coagulase-negative staphylococci

Differences in microbiological aetiology of HA-BSI were noted across the three groups (Fig. 2). The most frequent bacterial and fungal species isolated from each group are presented in Table 3).

**Table 3:** Most frequent causative organisms of HA-BSI

Haematology *n* isolates = 378 *n* episodes = 299			Oncology *n* isolates = 120 *n* episodes = 91			General medicine *n* isolates = 324 *n* episodes = 279		
	n	(%)		n	(%)		n	(%)
*Staphylococcus epidermidis*	90	(24)	*Escherichia coli*	24	(20)	*Escherichia coli*	54	(17)
*Escherichia coli*	53	(14)	*Staphylococcus epidermidis*	11	(9)	*Staphylococcus aureus*	41	(13)
*Streptococcus mitis/oralis*	51	(14)	*Pseudomonas aeruginosa*	7	(4)	*Staphylococcus epidermidis*	37	(11)
*Enterococcus faecalis*	20	(5)	*Staphylococcus aureus*	7	(6)	*Klebsiella pneumoniae*	31	(10)
*Enterococcus faecium*	19	(5)	*Enterococcus faecalis*	7	(6)	*Pseudomonas aeruginosa*	22	(7)
*Pseudomonas aeruginosa*	15	(4)	*Klebsiella pneumoniae*	7	(6)	*Klebsiella aerogenes*	12	(4)
*Klebsiella pneumoniae*	10	(3)	*Enterococcus faecium*	6	(5)	*Enterobacter cloacae complex*	12	(4)
*Granulicatella adiacens*	9	(3)	*Streptococcus anginosis*	4	(3)	*Candida albicans*	10	(3)
*Klebsiella oxytoca*	7	(2)	*Enterobacter cloacae complex*	3	(3)	*Enterococcus faecium*	10	(3)
*Staphylococcus haemolyticus*	6	(2)	*Bacteroides fragilis*	3	(3)	*Enterococcus faecalis*	9	(3)
			*Staphylococcus haemolyticus*	3	(3)			
			*Streptococcus mitis/oralis*	3	(3)			
			*Klebsiella oxytoca*	3	(3)			
			*Streptococcus parasanguinus*	3	(3)			
Others	96	(24)	Others	24	(20)	Others	86	(27)

*Results displayed as % of isolates

HA-BSI were polymicrobial in 63/299 (21%) episodes in haematology patients, 15/91 (16%) episodes in oncology patients, and 34/279 (12%) episodes in general medical patients (*p* = 0.02). Microbiology of polymicrobial and monomicrobial HA-BSI is presented in Supplementary Fig. 2.

In haematology patients, the majority of HA-BSI isolates were low virulence Gram-positive organisms (232/378, 61%), primarily coagulase-negative staphylococci (CoNS, 97/378, 26%), viridans group streptococci (71/378, 18%), and enterococci (40/378, 11%). Gram-negative organisms accounted for only 121/278 (32%) of all isolated organisms.

Compared to haematology patients, Gram-negative organisms were more commonly isolated in oncology patients (54/120 isolates, 45%) and in general medical patients (168/324 isolates 52%) and this difference was statistically significant (*p* < 0.01). Most Gram-negative isolates in these groups were Enterobacterales (oncology 44/120, 36%; general medical patients 138/324, 43%).

Hospital-acquired *Staphylococcus aureus* was very rarely isolated in haematology patients (1/299, 0.3% of HA-BSI episodes; zero of 45 CABSI), compared to oncology patients (7/91, 8% of HA-BSI; 3/20, 15% of CABSI) and general medical patients (42/279, 15% of HA-BSI; 13/52, 25% of all CABSI, *p* < 0.01 for all comparisons).

Hospital-acquired fungaemia was relatively uncommon, with no statistically significant difference between groups. Among all HA-BSI episodes, fungi were isolated in haematology patients in 8/299 (2.7%), in oncology patients 3/91 (3%), in general medical patients 17/279 (6%, *p* = 0.11). Among all CABSI episodes fungi were isolated in haematology patients in 2/45 (4%), in oncology patients 1/20 (5%) and general medical patients 0/52 (0%, *p* = 0.29). Most hospital-acquired fungaemia episodes were caused by *Candida albicans*,* Nakaseomyces glabratus *(formerly *Candida glabrata)*, or *Candida dublinensis *(23/30 unique fungal isolates, 75%). The remaining fungal species were *Rhodotorula mucilaginosa* (4 episodes, all in haematology patients), *Lomentospora prolificans* (2 episodes in haematology patients) and *Saccharomyces cerevisiae* (one episode in a general medical patient).

Antimicrobial resistance patterns are reported in Table 4. The proportion of Enterobacterales harbouring extended spectrum beta-lactamase (ESBL) was similar across all groups (haematology 15%; oncology 7%; general medicine 14%, *p* = 0.19), and in keeping with overall ESBL rates in our institution. Other antibiotic resistant organisms including CPE, VRE, and MDR-PsA were very uncommon.

**Table 4:** Resistance patterns of HA-BSI

	Haematology *n* isolates = 378 *n* episodes = 299			Oncology *n* isolates = 120 *n* episodes = 91			General medicine *n* isolates = 324 *n* episodes = 279		
	n	% HA-BSI	% eligible BSI	n	% HA-BSI	% eligible BSI	n	% HA-BSI	% eligible BSI
**Gram-negative**									
ESBL Enterobacterales	14	4%	15%	5	3%	7%	18	6%	14%
Carbapenemase-producing Enterobacteriaceae	0	-	-	0	-	-	0	-	-
Multidrug resistant Pseudomonas aeruginosa	3	1%	20%	2	1%	25%	2	1%	10%
Carbapenem resistant Pseudomonas aeruginosa	0	0%	0%	0	0%	0%	3	1%	14%
**Gram-Positive**									
MRSA	0	0%	0%	0	0%	0%	5	2%	12%
VRE	1	< 1%	2%	0	0%	0%	0	0%	0%

HA-BSI Hospital-acquired bloodstream infection Eligible BSI = causative organisms for which resistance profile is relevant (e.g. MRSA among *Staphylococcus aureus*)

ESBL extended spectrum beta-lactamase MRSA methicillin resistant *Staphylococcus aureus* VRE Vancomycin resistant enterococci

Multi-drug resistant Pseudomonas aeruginosa = resistant to ≥ 1 agent in > 3 antimicrobial categories. No multi-resistant lactose non-fermenters other than *Pseudomonas spp.* were isolated

### Microbiology of CABSI

Microbiology of CABSI is reported in Fig. 2. Among haematology patients, the majority of CABSI isolates were CoNS (43/52, 83%). In oncology patients, Gram-negative organisms were the isolated most frequently (16/28, 57%). For general medical patients, common CABSI isolates were CoNS (21/54, 39%), Gram-negative organisms (18/54, 33%) and *S. aureus* (13/54, 24%).

We performed two sensitivity analyses first including only microbiologically confirmed CABSI (ECDC CRI-3 definition), and secondly excluding polymicrobial infections. The distribution of CABSI organisms remained similar across all groups in these analyses (Supplementary Fig. 3).

## Discussion

We performed a retrospective cohort study of haematology, (acute leukaemia and allogeneic stem cell transplant recipients), oncology and general medicine patients between 2017 and 2022 in a hospital network where routine quinolone prophylaxis is not used. We identified 669 HA-BSI and compared the characteristics of those occurring in haematology, oncology, and general medical patients. We observed marked differences in the source and microbiology of infections, highlighting important differences in optimal management and prevention. There is a currently a lack of detailed HA-BSI surveillance data reported in cancer patients, and we are not aware of any similar studies comparing HA-BSI and CABSI characteristics between cancer types and the general hospital population.

In patients with acute leukaemia and allogeneic stem cell transplant recipients not receiving quinolone prophylaxis, incidence of HA-BSI was highest, and most BSI were of unknown/other source, but the proportion caused by high virulence organisms, including Gram-negative bacteria and *Staphylococcus aureus*, was lowest. In contrast, patients with solid cancers and general medical patients had lower HA-BSI incidence, higher rates of secondary BSI, and substantially higher proportion of infections caused by virulent Gram-negative bacteria and *S. aureus.* Antibiotic resistance was minimal across all groups; 6–15% of all Enterobacterales harboured an ESBL, mirroring the broader hospital and community antibiogram, while other antibiotic resistant organisms (e.g. CPE/CRE, VRE, MDR-PsA) were very uncommon.

The high rates of BSI of “unknown/other source” in haematology patients have important clinical and surveillance implications. A proportion of primary infections of “unknown/other” source represent translocation of endogenous flora across an inflamed oral or gastrointestinal mucosa, also known as mucosal barrier injury bloodstream infections (MBI-BSI). However, MBI-BSI are poorly defined. The North American Centers for Disease Control and Surveillance (CDC) definition of MBI-BSI attributes any primary BSI to mucosal injury if the causative pathogen is an “enteric” organism (presumed mouth or bowel flora), occurring in a patient with neutropenia or severe intestinal graft versus host disease. This classification is based on artificial classification of “enteric” organisms and is prone to inaccuracy; for example, when sufficient microbiological data are available, up to one quarter of these infections are reclassified as CABSI [4–7]. Despite these limitations, MBI-BSI are excluded from surveillance in many jurisdictions, including the United States [3], precluding detailed analysis of the source, preventability, and longitudinal trends of these infections.

ECDC definitions do not specifically define MBI-BSI. Therefore, using the ECDC definitions applied in this study, MBI-BSI are captured, but are classified with “infections of unknown source”, potentially combining clinically distinct infections under one classification. We identified that these BSI of “unknown/other” source represent 75% of all HA-BSI in haematology patients. These findings indicate a need to extend and refine surveillance definitions to better characterise these infections [4, 5]. While comparisons to previous data are difficult, due to variation in surveillance and research definitions, one cohort study in Spain reported similarly high rates of “unknown” infection at 57% [8]. Importantly, the preventable fraction of MBI-BSI is not known, and epidemiological trends are not well characterised [15]. MBI-BSI have been shown to comparable outcomes to other HA-BSI, and therefore warrant further study [15].

We observed a low proportion of Gram-negative BSI in acute leukaemia and stem cell transplant patients, in the absence of quinolone prophylaxis. This is unexpected; previous single-centre reports from cancer units in Spain, Italy, Australia, and Korea have reported higher frequency of Gram-negative organisms in haematology patients, approaching 50% of all BSI [8, 16–18]. Low rates of Gram-negative infections in our haematology patients is unlikely to be explained by global differences in population or hospital epidemiology, given that Gram-negative BSI occurred more commonly in solid tumour and general medical patients in our centre.

This finding is clinically important. Patients with acute leukaemia or undergoing stem cell transplantation are generally considered to be at very high risk of Gram-negative BSI. Quinolone prophylaxis has previously been shown to reduce risk of BSI, but carries risks of increased rates of antimicrobial resistance and has not shown clear mortality benefit [10]. Quinolone prophylaxis remains widespread in the United states and some parts of Europe, but is not recommended in some jurisdictions and is an ongoing topic of debate [10, 19]. We showed that in patients not receiving quinolone prophylaxis, Gram-negative BSI represent a minority of infections, supporting the choice not to routinely prescribe quinolones.

In addition, non-pharmacological prevention strategies for Gram-negative BSI may be important, but have not been widely studied [20]. The low frequency of Gram-negative BSI in our centre compared to previous reports warrants further investigation to identify modifiable risk factors. Potential contributing factors include infection prevention practices, antimicrobial stewardship, mouth care, selective oral decontamination, and the hospital and ward environment, (including architectural design), as well as choice of immunosuppression and conditioning regimens.

We also observed strikingly low rates of *S. aureus* BSI in the leukaemia and stem cell transplantation group. Other centres have reported higher frequency of *S. aureus* BSI (6–12% of all BSI) in haematological cancer patients [8, 16–18]. However, previous Swiss data from the Swiss National Transplant Cohort report similar microbiology to the present study, with very low proportion of *S. aureus* BSI in bone marrow transplant patients [21]. Low rates of *S. aureus* BSI in heavily immunosuppressed haematology patients may relate to the exacting infection prevention and control measures implemented on haematology wards, use of selective oral decontamination, or to changes in the microbiome in patients receiving high-intensity chemotherapy [22, 23]. Again, further dedicated study may identify targets for prevention of high virulence BSI. Conversely, high rates of BSI caused by skin commensals warrant further investigation.

Differences in catheter use may also contribute to the variation in microbiology and source of infection across patient groups. Non-tunnelled CVC use was substantially more frequent in haematology compared to TIVAD or PVC, and may be associated with lower rates of Gram-negative and *S. aureus* infections [24, 25]. The relative risks and benefits of different catheter types in cancer patients is an important area of ongoing study.

Compared to haematology patients, we observed a high proportion of Gram-negative infections in (solid tumour) oncology and general medical patients, including in CABSI. Gram-negative CABSI were frequent, even when analysis was limited to microbiologically confirmed catheter infections. Catheters may be overlooked at the source of these infections; a high index of suspicion for CABSI and aggressive empiric therapy may be warranted in this group.

Finally, fungaemia accounted for 3% of all HA-BSI in our haematology and oncology cohort. This is comparable to single centre reports in cancer patients in Spain and Australia [8, 18]. However, compared to these previous reports, we observed a higher proportion of fungaemia in haematology patients caused by *Nakaseomyces* glabratus and rare fungi *Rhodotorula mucilaginosa* and *Lomentospora prolificans.* This is consistent with dedicated studies of invasive fungal infection in haematology patients, which have shown increasing in breakthrough infection with rare fungi in the setting of mould-active antifungal prophylaxis [26]. These infections require a high index of suspicion and aggressive treatment.

Regarding antimicrobial susceptibility, we observed very low rates of carbapenem resistant Gram-negative infections, and vancomycin-resistant enterococci, and modest rates (around 15%) of ESBL Enterobacterales across all patient groups. Comparable rates of antimicrobial resistance in the ICU setting in Switzerland have been previously reported [27]. In general, haemato-oncology patients are at high risk of emergent antimicrobial resistance, with risk factors such as prolonged hospitalisation and antibiotic exposure. It is reassuring that despite these risks, resistance rates in haemato-oncology patients in our centre do not exceed those in the general hospital population. Antimicrobial resistance in our institution is lower than in many parts of Europe; for example, an Italian cohort published in 2015 reported 35% of Klebsiella pneumoniae isolates resistant to carbapenems [16, 28]. However, reporting of antimicrobial resistance is limited by inconsistency in surveillance, and resistance patterns are rapidly evolving; therefore, dedicated study is needed to assess the burden of antimicrobial resistance in haemato-oncology patients [28–30].

This study has several limitations. These are single centre data. We analysed only observational surveillance data and had limited access to clinical data, including details of underlying cancer type, patient characteristics, and presence of neutropenia, all of which are likely to contribute to HA-BSI risk. This limits the analysis in several important ways.

Firstly, microbiology of HA-BSI is likely to differ between individual cancer groups within the haematology and oncology groups. In particular, epidemiology of HA-BSI in lymphoma patients may differ from solid tumours, and this is not captured in this analysis. In our institution, the oncology unit manages patients with lymphoma (provided they are not stem cell transplant recipients), together with patients with solid tumours, and these patients are grouped together in our surveillance data. The haematology unit cares for patients with acute leukaemia and allogeneic stem cell transplant recipients. Management of lymphoma under oncology units is seen in some centres in Europe, with lymphoma management under haematologist slightly more common overall [31]. Some previous studies of HA-BSI epidemiology in haematology patients have included lymphoma and acute leukaemia patients groups together, although lymphoma patients accounted for a minority (around 20%) of included patients [16, 18]. Other authors have reported on HA-BSI in “haematology” patients without specifying underlying diagnoses [8]. In short, patients with lymphoma are poorly captured in existing analyses of hospital-acquired infection, including the present analysis, and dedicated study is needed. Furthermore, we do not include any autologous stem cell transplant recipients in this study, and our findings may not be generalisable to this group.

In contrast, acute leukaemia and stem cell transplant recipients have been grouped together in previous observational and interventional trials relating of antimicrobial prophylaxis, diagnosis and management of infections [10, 32–34]. These patients typically receive very high-intensity chemotherapy, experience prolonged neutropenia and profound immunosuppression, and have been shown to have higher risk of hospital-acquired infection than patients with lymphoma [35]. The majority of all clinical data on quinolone prophylaxis comes from patients with acute leukaemia and allogeneic stem cell transplant [32]. We believe these patients represent a distinct clinical cohort and our findings suggest HA-BSI epidemiology differs in this group compared to other cancer patients.

Secondly, in the absence of detailed clinical data we are unable to comment on underlying risk factors for HA-BSI or compare patients who developed HA-BSI with those who did not. We also did not have access to data describing the presence or absence of neutropenia, in order to assess which infections met MBI-BSI criteria according to alternative surveillance definitions. This limits comparisons with other jurisdictions (such as the United States).

Finally, we had only limited antimicrobial susceptibility data. Detailed analysis of antimicrobial resistance would be valuable, particularly quinolone susceptibility rates in the absence of prophylaxis. Centres using routing quinolone prophylaxis have reported very high rates of quinolone resistance, as well as increased rates of resistance to other agents in patients who develop BSI [36, 37]. In addition, given high rates of coagulase-negative staphylococcal infections, an assessment of antimicrobial susceptibility in this group would be valuable.

## Conclusions

In a large cohort study, we identified marked differences in the epidemiology of HA-BSI between haematology patients with acute leukaemia and allogeneic stem cell transplant, oncology patients with solid tumours or lymphoma, and the general hospital population. Haematology patients had proportionally fewer infections with virulent Gram-negative organisms and *S. aureus* compared to solid tumour/lymphoma and general medical patients, despite no routine use of quinolone prophylaxis. Further study to identify preventative strategies that contribute to these findings are needed. Active surveillance of all HA-BSI may help identify targets for prevention in cancer patients.

## Electronic supplementary material

Below is the link to the electronic supplementary material.

Supplementary Material 1

## Data Availability

No datasets were generated or analysed during the current study.
