# Supplementary material for: Epidemiology of hospital-acquired bloodstream infections in haemato-oncology patients in Geneva, Switzerland
Source: Infection. 2025 Apr 9;53(5):1929–39. doi: 10.1007/s15010-025-02524-w (PMC12460527; doi:10.1007/s15010-025-02524-w)
Supplement: Supplementary file 1 — Supplementary Material 1 [file 15010_2025_2524_MOESM1_ESM.docx]

**Supplementary material: Epidemiology of hospital acquired bloodstream infections in haemato-oncology patients**

**Supplementary methods: Definitions**

Hospital acquired bloodstream infections (HA-BSI) were defined according to European Centre for Disease Control (ECDC) definitions (1, 2) as culture of a pathogen from blood culture 48 hours after the beginning of the current admission or meeting one of the following criteria: readmission <10 days after discharge from a previous admission; care in an ambulatory dialysis or haemato-oncology unit <30 days prior; infection related to a surgery <30 days prior; or infection related to surgery with implantation of a prosthesis <365 days prior. Common skin contaminants isolated from blood cultures were included only if the patient had at least 1 sign or symptom infection (e.g. fever >38^o^C, chills, hypotension) and 2 positive blood culture results from 2 separate blood samples (i.e. two blood draws from separate sites and/or separate times) within 48 hours. Common skin contaminants were defined according ECDC or National Healthcare Safety Network (NHSN) official list (e.g., coagulase–negative staphylococci [CoNS], *Corynebacterium* spp., *Bacillus* spp., *Micrococcus* spp., and *Propionibacterium* spp.) (3)

HA-BSI were classified as either catheter-associated bloodstream infection (CABSI), BSI secondary to infection at another site, or BSI unknown source using ECDC definitions. We defined CABSI according to either ECDC Catheter Related Infection criteria (CRI3), or ECDC Hospital Acquired Bloodstream Infection (Central/Peripheral Catheter Origin) criteria, (ECDC-C-CVC/P). The CRI3 definition requires microbiological confirmation of a line source of infection demonstrated by positive blood culture <48 hours before or after catheter removal and the same microorganism isolated from a quantitative catheter tip culture of greater than 103 colony forming units (CFU)/mL, or semiquantitative culture of >15 CFU/ml; the same microorganism isolated in a culture from pus collected from a catheter site; or differential delay of positivity of simultaneously drawn blood cultures, with a CVC blood sample culture positive two hours or more before peripheral blood culture. The C-CVC/P definitions require isolation of the same organism from peripheral blood and central/peripheral catheter, or improvement of symptoms within 48 hours of removal of a catheter, in the absence of another source. ECDC definitions do not require a minimum catheter dwell time for attribution of catheter source.

**Supplementary methods: institutional catheter care procedures**

Institutional recommendations for preventing catheter-associated infections include the following: (i) maximal sterile barrier precaution of clinicians at the time of insertion (centrally dwelling catheters only); (ii) alcohol-containing 2% CHG for skin antisepsis prior to catheter insertion; (iii) the selection of site of insertion and utilization of ultrasound guidance according to proceduralists discretion; (iv) semipermeable transparent dressings without antiseptic impregnation are used; (v) soiled, leaking, or wet dressings are immediately changed. Impregnated CHG dressings, CHG bathing, disinfecting caps are not routinely used.

In 2021-2022 a local intervention was undertaken, in which a policy of clinically-indicated PVC replacement was trialed for 18 months. Outside this period, institution policy required routine replacement of PVC every 96 hours (3).

**Supplementary references**

1. European Centre for Disease Prevention and Control (ECDC). Surveillance of healthcare-associated infections and prevention indicators in European intensive care units. 2017 Available from: <https://ecdc.europa.eu/sites/portal/files/documents/HAI-Net-ICU-protocol-v2.2_0.pdf>

2. European Centre for Disease Prevention and Control (ECDC), Point prevalence survey of healthcare- associated infections and antimicrobial use in European acute care hospitals – protocol version 6.1. 2022 Available from:<https://www.ecdc.europa.eu/sites/default/files/documents/antimicrobial-use-healthcare-associated-infections-point-prevalence-survey-version6-1.pdf>

3. Centers for Disease Control and Prevention. CDC/NHSN Master organism list. 2023; Available from: <https://www.cdc.gov/nhsn/psc/bsi/index.html>.

4. Buetti, N., M. Abbas, D. Pittet, M.E. de Kraker, D. Teixeira, M.-N. Chraiti, et al., Comparison of routine replacement with clinically indicated replacement of peripheral intravenous catheters. JAMA Internal Medicine, 2021. 181(11): p. 1471-1478.

**Supplementary figure 1:** Included episodes


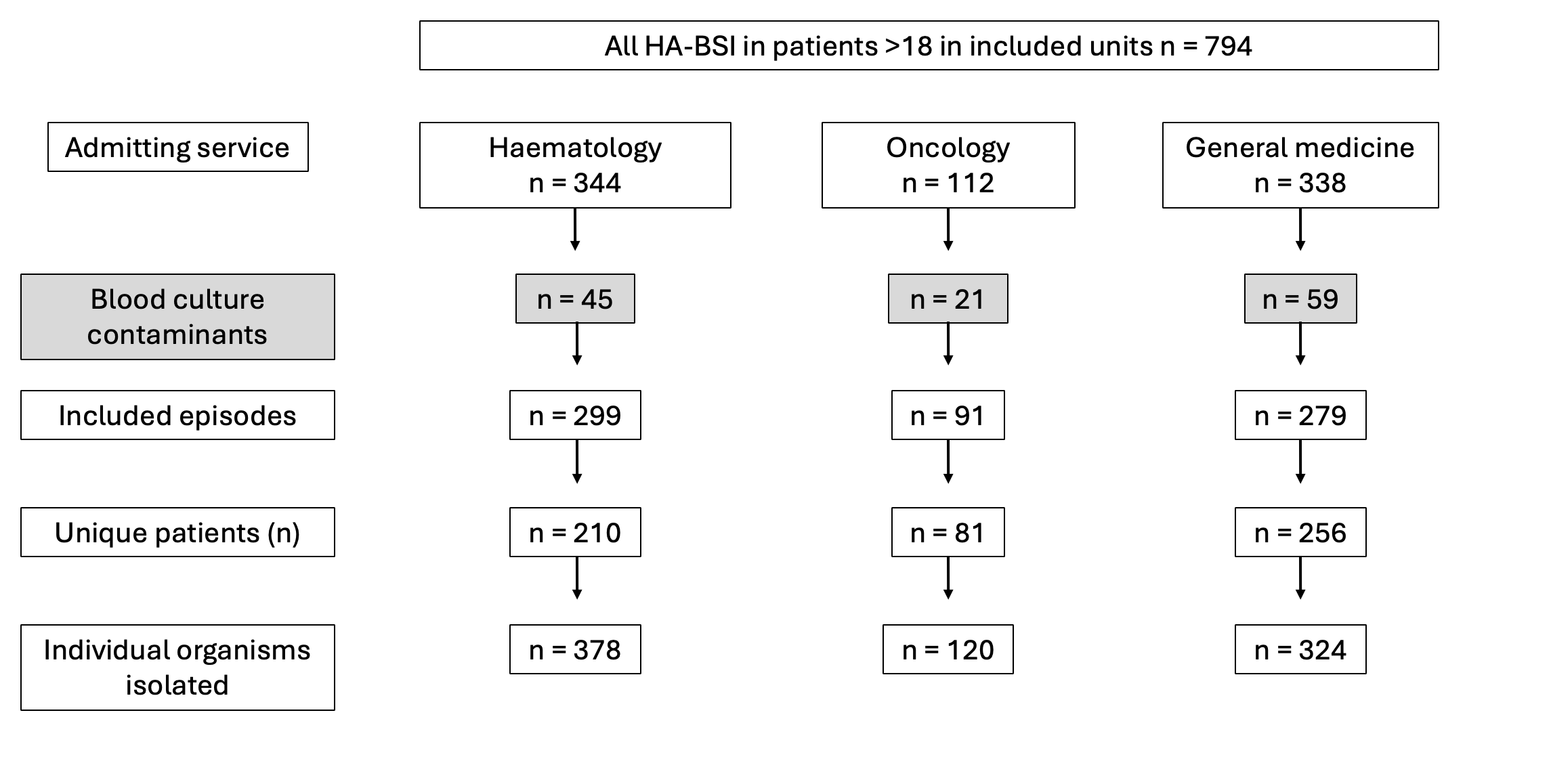


Multiple episodes of HA-BSI, defined by two or more HA-BSI episodes during the study period occurring more than 14 days apart in the same individual patients, occurred in 50/210 (24%) individual haematology patients, 9/91 (13%) individual oncology patients and 18/256 (8%) individual general medical patients (p <0.01)

HA-BSI were polymicrobial in 63/299 (21%) episodes in haematology patients, 15/91 (16%) oncology patients, and 34/279 (12%) general medical patients (p = 0.02).

**
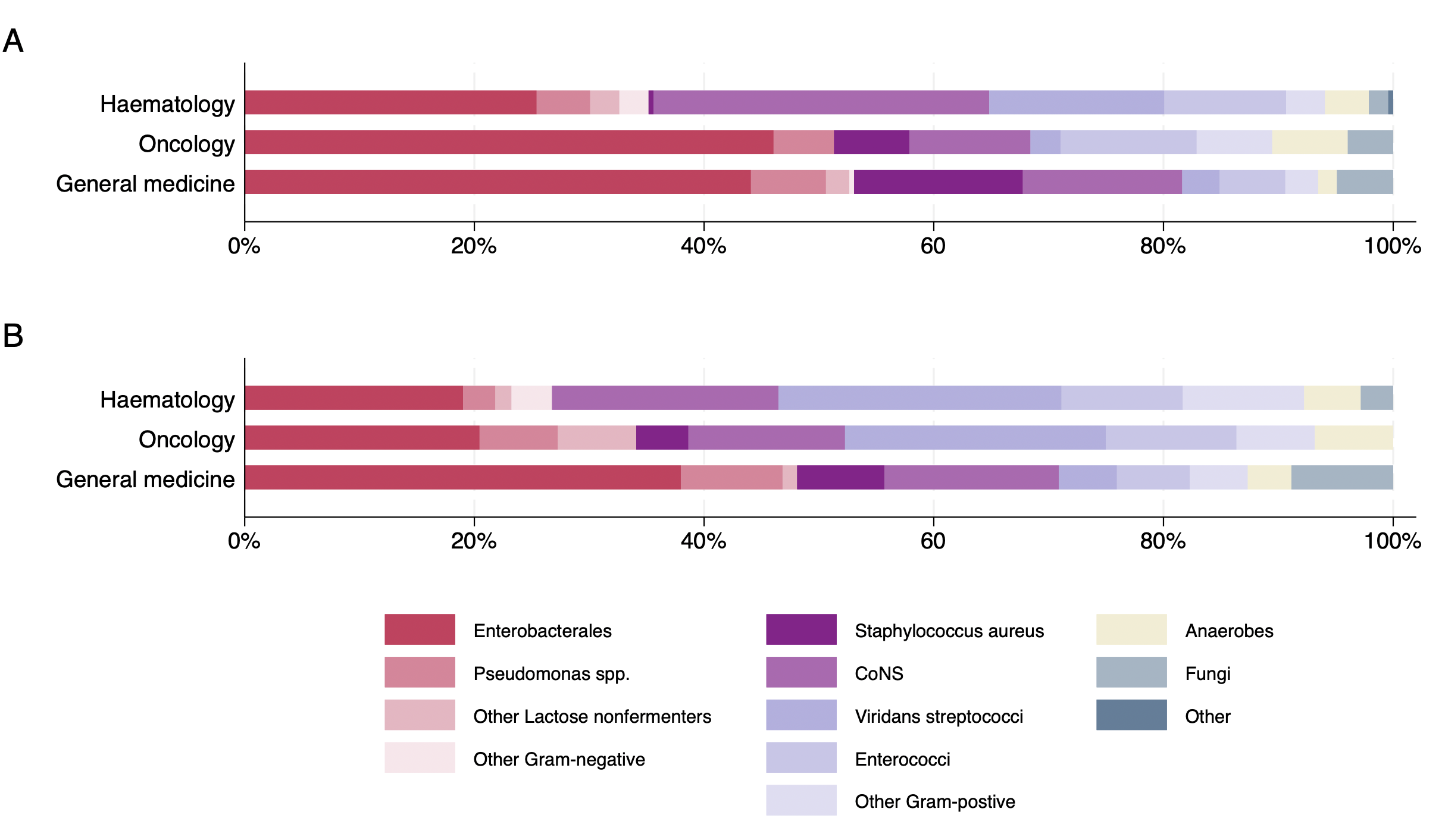
Supplementary figure 2:** Microbiology of polymicrobial hospital-acquired bloodstream infections: unique organisms isolated

**A:** Monomicrobial HA-BSI n = 557 unique isolates **B:** Polymicrobial HABSI n = 265 unique isolates

CoNS: coagulase negative staphylococci

**
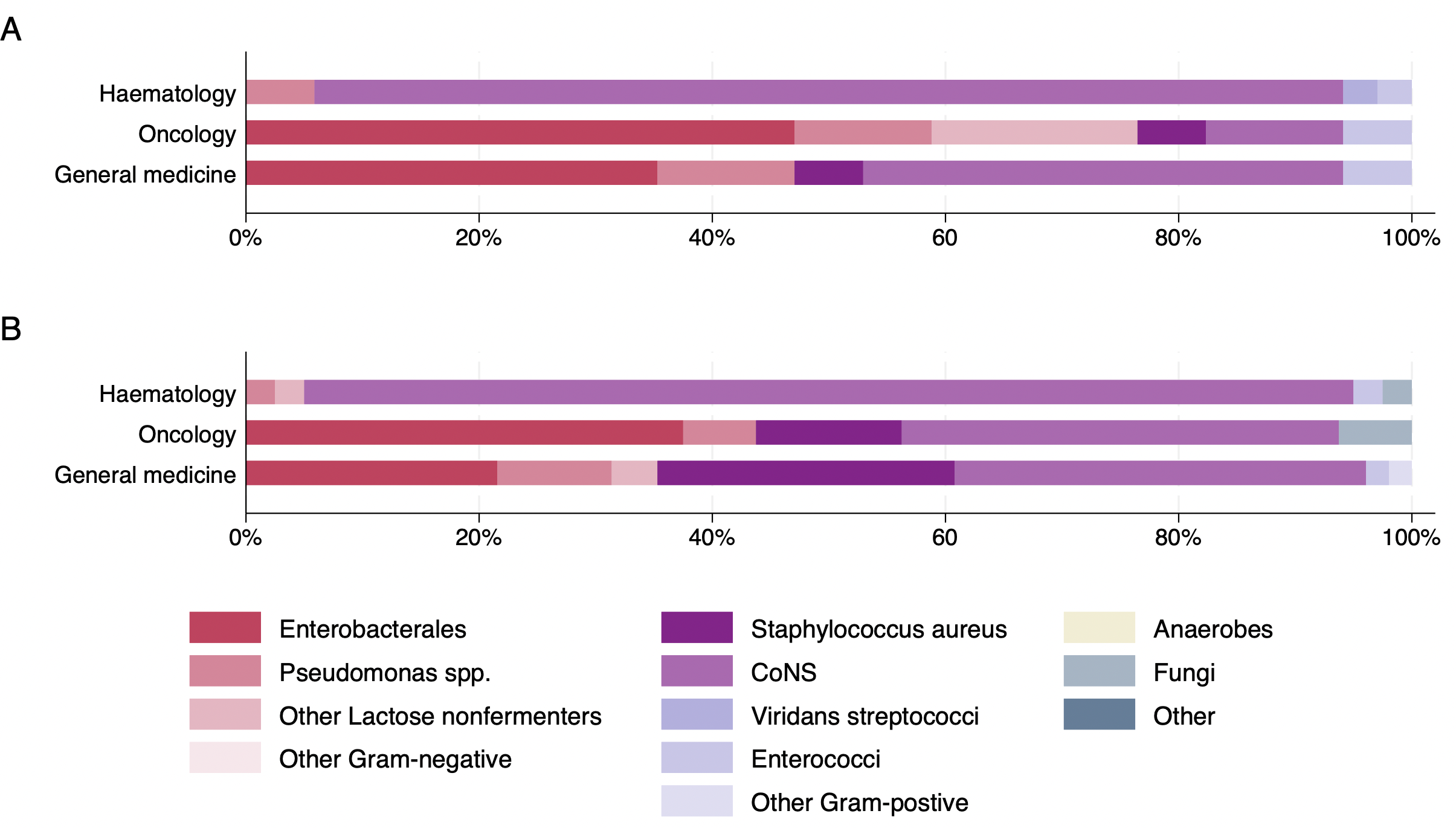
**

**Supplementary figure 3:** Microbiology of catheter associated bloodstream infections sensitivity analysis

**A:** Microbiologically confirmed CABSI (ECDC CRI3 definition) n = 58 **B:** Polymicrobial CABSI excluded n = 107

CoNS: coagulase negative staphylococci

p <0.01 for comparison between groups
